# Identification of pathological transcription in autosomal dominant polycystic kidney disease epithelia

**DOI:** 10.1038/s41598-021-94442-8

**Published:** 2021-07-23

**Authors:** Sebastian Friedrich, Hannah Müller, Caroline Riesterer, Hannah Schüller, Katja Friedrich, Carlotta Leonie Wörner, Tilman Busch, Amandine Viau, E. Wolfgang Kuehn, Michael Köttgen, Alexis Hofherr

**Affiliations:** 1 Renal Division, Department of Medicine, Medical Center, Faculty of Medicine, University of Freiburg, Hugstetterstrasse 55, 79106 Freiburg, Germany; 2 Imagine Institute, Laboratory of Hereditary Kidney Diseases, INSERM UMR 1163, Université de Paris, 75015 Paris, France; 3 CIBSS – Centre for Integrative Biological Signalling Studies, Freiburg, Germany; 4 Present Address: Department of General Pediatrics, Adolescent Medicine and Neonatology, Center for Pediatrics, Medical Center, Faculty of Medicine, University of Freiburg, Mathildenstrasse 1, 79106 Freiburg, Germany

**Keywords:** Kidney diseases, Kidney, Molecular medicine, Pathogenesis

## Abstract

Autosomal dominant polycystic kidney disease (ADPKD) affects more than 12 million people worldwide. Mutations in PKD1 and PKD2 cause cyst formation through unknown mechanisms. To unravel the pathogenic mechanisms in ADPKD, multiple studies have investigated transcriptional mis-regulation in cystic kidneys from patients and mouse models, and numerous dysregulated genes and pathways have been described. Yet, the concordance between studies has been rather limited. Furthermore, the cellular and genetic diversity in cystic kidneys has hampered the identification of mis-expressed genes in kidney epithelial cells with homozygous PKD mutations, which are critical to identify polycystin-dependent pathways. Here we performed transcriptomic analyses of Pkd1- and Pkd2-deficient mIMCD3 kidney epithelial cells followed by a meta-analysis to integrate all published ADPKD transcriptomic data sets. Based on the hypothesis that Pkd1 and Pkd2 operate in a common pathway, we first determined transcripts that are differentially regulated by both genes. RNA sequencing of genome-edited ADPKD kidney epithelial cells identified 178 genes that are concordantly regulated by Pkd1 and Pkd2. Subsequent integration of existing transcriptomic studies confirmed 31 previously described genes and identified 61 novel genes regulated by Pkd1 and Pkd2. Cluster analyses then linked Pkd1 and Pkd2 to mRNA splicing, specific factors of epithelial mesenchymal transition, post-translational protein modification and epithelial cell differentiation, including CD34, CDH2, CSF2RA, DLX5, HOXC9, PIK3R1, PLCB1 and TLR6. Taken together, this model-based integrative analysis of transcriptomic alterations in ADPKD annotated a conserved core transcriptomic profile and identified novel candidate genes for further experimental studies.

## Introduction

Autosomal dominant polycystic kidney disease (ADPKD) is the most common hereditary nephropathy in humans^1^. Most patients with ADPKD are born healthy, but progressive cystic transformation of both kidneys induces a continuous decline in renal function leading to kidney failure^1^. Mutations in two genes, *PKD1* and *PKD2*, cause ADPKD^2,3^. The protein products of these genes, polycystin-1 (PC-1) and transient receptor potential channel polycystin-2 (TRPP2) form a receptor-ion channel complex that is essential to establish renal tubular morphology and prevent cystogenesis^1^.

Many cellular signaling pathways have been implicated in the pathogenesis of ADPKD, including Wnt, mTOR, JAK/STAT and Hippo^4–8^. Yet, the precise molecular function of PC-1/TRPP2 and the signaling pathways critical for disease initiation have remained unclear^9^. Unbiased, discovery-based approaches offer unique opportunities to address these key questions of ADPKD pathology. RNA sequencing (RNA-seq) technology has made large scale analysis of gene expression a powerful approach to systematically investigate disease mechanisms^10,11^. Consequently, multiple studies have explored transcription in cystic kidneys from Pkd-deficient mice and ADPKD patients. These studies have reported a large number of differentially expressed genes in grossly cystic samples^12–19^. However, the concordance between studies for specific gene sets has been low^15,19^. This complex variability is likely to be explained by differences in experimental design, tissue sampling and model system^19^. Hence, transcriptomic profiling of ADPKD has confirmed broad cellular alterations in cystic tissue, but has provided limited novel insights into PKD-dependent tubule-cell-specific factors causing cystic kidney disease.

A key challenge of specific profiling of *PKD1*- and *PKD2*-dependent transcription has been the genetic mosaicism of organisms with ADPKD. In ADPKD, cysts arise from individual renal epithelial cells upon somatic loss of heterozygosity for *PKD1* or *PKD2*^20^. This focal process has been modeled in mice by homozygous Pkd inactivation in kidney tubular epithelial cells. However, in both cases only a small fraction of renal cells harbor the homozygous PKD mutations required to trigger cystogenesis. The majority of cells in polycystic kidneys are still heterozygous for PKD gene mutations—or wildtype in the case of tubule-specific knockout mice. Furthermore, due to secondary processes, such as proliferation of cyst epithelia, inflammation and fibrosis, cystic kidneys show considerable secondary changes in cellular composition^21^. These processes contribute to disease progression, but further confound the transcriptomic profiling of homozygous mutant tubular cells at cyst initiation, a prerequisite to gain insights into PKD gene function.

**Figure 1:**
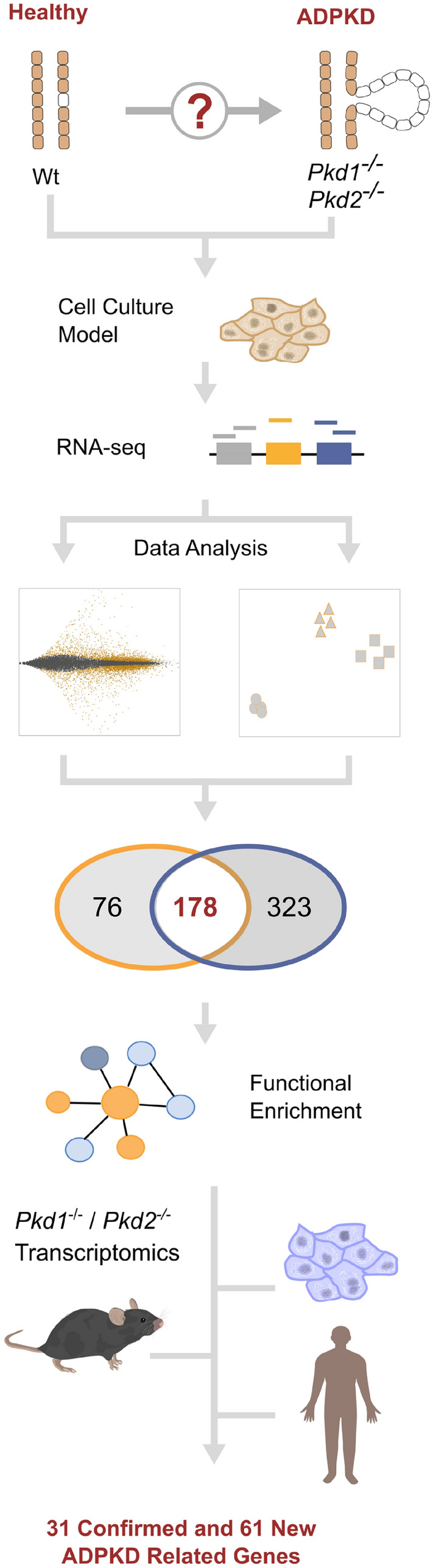
Model-guided meta-analysis of ADPKD-responsive transcription identified consistent mis-regulation of 31 known and 61 new genes. Unbiased analysis of Pkd1- and Pkd2-dependent mRNA transcription in genome-edited renal tubular epithelial mIMCD3 ADPKD cells provided rich data with minimal variability. Core Pkd-dependent transcription was delineated by statistical analysis of differential gene expression and exploratory data simplification by principal component analysis. Function-based cluster analysis of identified regulation isolated key pathways caused by loss of Pkd genes. ADPKD disease-relevance was investigated by model-guided meta-analysis of Pkd1- and Pkd2-deficient cells and mice, as well as human PKD1^−/−^ kidneys. Ultimately, integrative transcriptomics confirmed 31 previously described transcriptional regulations and identified 61 new genes transcriptionally regulated by both, PKD1 and PKD2.

Here, we investigate cell-autonomous ADPKD-dependent transcriptional changes based on two ground truths: (1) mutations in *PKD1* and *PKD2* cause ADPKD^2,3^; and (2) renal cystogenesis is initiated in PKD-deficient tubular epithelial cells^20^. First, we characterized transcriptomic profiles of genetically engineered, clonal ADPKD cells in a well-controlled, reductionist approach. RNA-seq of *Pkd1-* and *Pkd2*-deficient mouse inner medullary collecting duct 3 (mIMCD3) epithelial cells identified a core ADPKD data set of 178 reproducible and concordantly regulated genes. Using this core data set, we next performed a cross-species meta-analysis to integrate the wealth of transcriptomic data from *Pkd*-deficient animals and human ADPKD kidneys, which confirmed 31 highly consistent, previously described, transcriptionally regulated genes. Notably, we also identified 61 novel genes that are differentially expressed in *Pkd1* and *Pkd2* deficiency (Fig. 1). Gene ontology and functional pathway enrichment analyses linked specific factors of epithelial mesenchymal transition (EMT), post-translational protein modification and epithelial cell differentiation to ADPKD pathogenesis. This combination of cellular PKD models with transcriptomic data from cystic kidneys showed the power of integrative transcriptomics to validate candidate targets for in-depth studies of pathogenic mechanisms in ADPKD, including *CD34*, *CDH2*, *CSF2RA*, *DLX5*, *HOXC9*, *PIK3R1*, *PLCB1* and *TLR6*.

## Results

### Transcriptional characterization of ADPKD by deep RNA sequencing of Pkd1- and Pkd2-deficient renal tubular cells

Mouse inner medullary collecting duct 3 (mIMCD3) cells represent a well-established, differentiated in vitro model of the distal nephron^22^. mIMCD3 cells are highly differentiated, phenotypically stable and show low heterogeneity, with preserved apico-basolateral polarity, formation of primary cilia, collecting duct-like transport activity and tubule formation in 3D culture^22–25^. This enables stringent analyses of gene-dependent transcription in a controlled environment, free of systemic variations (Figure S1a)^26^.

Analysis of active gene regulatory networks as major molecular determinants of cell-type identity further confirmed the kidney transcriptional signature of mIMCD3 cells (Figure S1b)^27^. For the targeted evaluation of ADPKD-dependent cellular pathophysiology, we here compared wild-type mIMCD3 cells with constitutive *Pkd1*- and *Pkd2*-deficient cell lines^28^.

**Figure 2:**
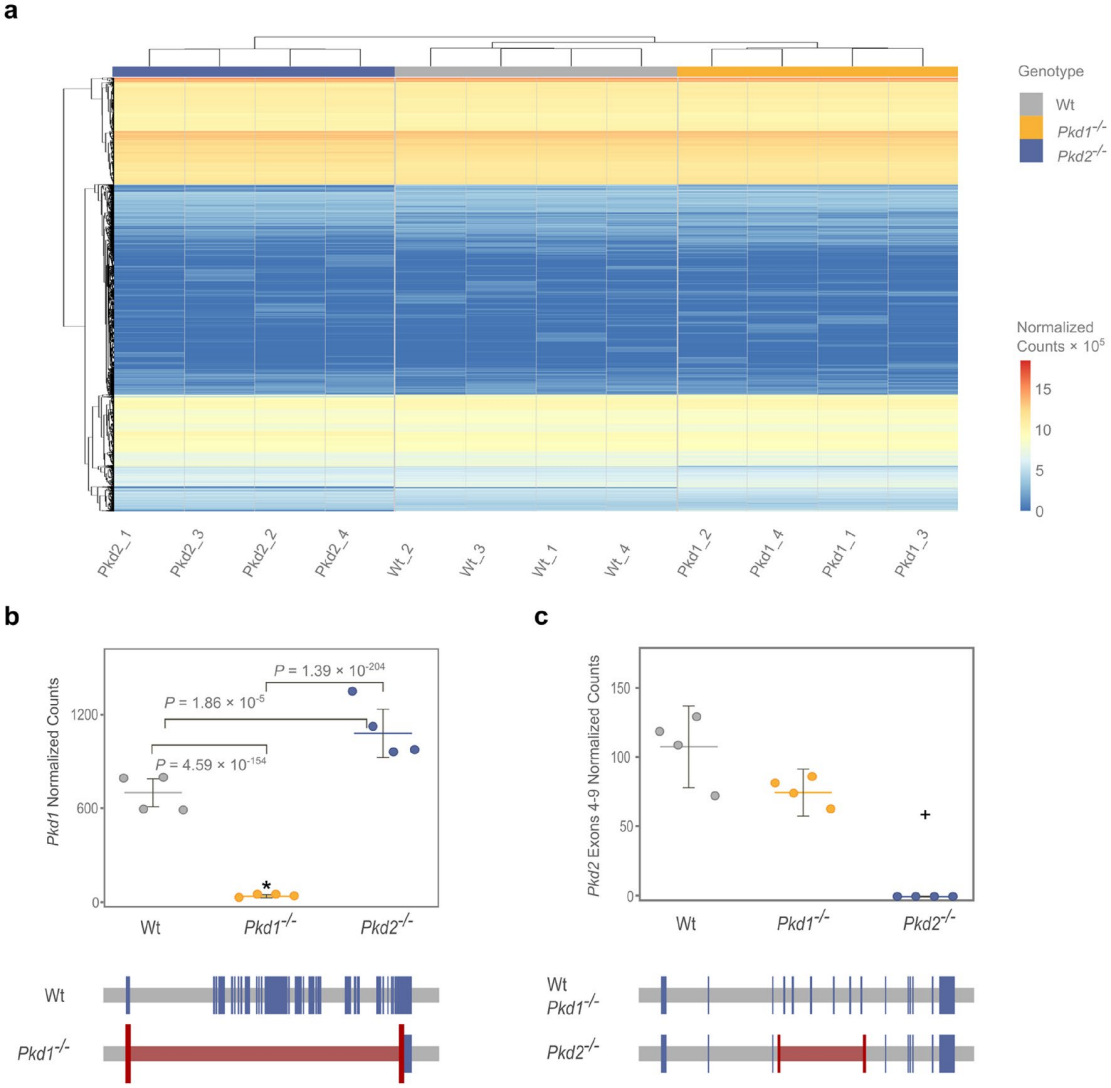
Transcriptomic profiling of genetically engineered ADPKD cells showed significant interdependence of Pkd1 and Pkd2 mRNA expression. (**a**) Unsupervised hierarchical clustering showed low variability and genotype-dependent grouping of mIMCD3 Wt, Pkd1^−/−^ and Pkd2^−/−^ cells. (**b**) Pkd1 knockout was generated by a full genomic deletion. Loss of Pkd2 was associated with a significant transcriptional up-regulation of Pkd1 mRNA (782.25 [SEM = 97] mean normalized counts in wildtype vs. 1,209 [SEM = 175] in Pkd2^−/−^ cells). (**c**) Pkd2 knockout was based on genomic deletion of Pkd2 exons 4–9 and no counts were detected for these exons. + : adjusted p-values for single exons 4–9 were < 1.75 × 10^–51^ in Pkd2^−/−^ versus Wt and Pkd2^−/−^ versus Pkd1^−/−^ cells.

Repeated deep RNA-seq of wild-type, *Pkd1*^−/−^ and *Pkd2*^−/−^ cells yielded rich and highly consistent transcriptomic profiles (Fig. 2a). Sequencing depth, read distribution between samples, sample-to-sample-distances, dispersion of variance and histogram of p-values verified excellent quality of data (Figure S2a–e).

Collected RNA-seq data confirmed the genomic deletion of *Pkd1* and *Pkd2*, and showed significant mutual interdependence of *Pkd1* and *Pkd2* mRNA expression, indicating a transcriptional *Pkd1/2* feedback loop (Figs. 2b,c, S1c,d). Full gene deletion removed all *Pkd1* transcripts in mutant cells and was associated with a decrease in *Pkd2* expression (− 31.7%) (Figs. 2b,c, S1c). Deletion of *Pkd2*, on the other hand, was associated with a significant transcriptional up-regulation of *Pkd1* mRNA (+ 54.6%) (Fig. 2b,c). RNA-seq of *Pkd2*^−/−^ cells also confirmed that genomic deletion of chromosome 5, bases 104,478,281 – 104,490,878, removed *Pkd2* exons 4–9 (of a total 15) and, hence, TRPP2 channel transmembrane domains 2–6 (of a total 6), which generated biochemically TRPP2-deficient cells (Figs. 2c, S1c).

### Unbiased statistical integration of Pkd1- and Pkd2-dependent transcription identified a core ADPKD data set of 178 consistently regulated genes (CD_178_)

Mutations in *PKD1* and *PKD2* cause ADPKD^2,3^. However, to date, no comprehensive analysis of *PKD1*- and *PKD2*-dependent transcription in ADPKD has been reported; several studies have focused on *PKD1* and limited data is available on *PKD2*-responsive transcription^18,19^. We hypothesized that the integrative analysis of *PKD1-* and *PKD2-*deficiency may substantially improve the high-confidence identification of consistent transcriptional changes in ADPKD pathogenesis.

Technically, the low biological variability of wild-type, *Pkd1*^−/−^ and *Pkd2*^−/−^ mIMCD3 cells provided a sound basis to delineate ADPKD-specific renal tubular transcription (Figs. 2a, S2a–e). This was important, as gene expression is an inherently stochastic process and is known to vary even between cells of the same population^29^. In general, observed variations in gene expression can be decomposed as^30,31^:

$${\text{Var}}\left( {{\text{Expr}}} \right) \, = {\text{ Across}}\,{\text{group}}\,{\text{variability}} + {\text{Measurement}}\,{\text{error}} + {\text{Biological}}\,{\text{variability}}.$$

With *group variability* being here the variation in gene expression due to the three genotypes under consideration. *Measurement error* being rather low in next-generation RNA-seq^32^. And *biological variability* being in our experiments restricted to stochastic cell-to-cell variation; and, therefore, minimal in comparison to tissue or cell type variability in whole organ analyses^31^. Thus, our approach of repeated sampling, high data quality and model simplicity should minimize the confounding effects of measurement error and biological variability, focusing generated data on the primary outcome of interest: the estimated Pkd-dependent difference in gene expression between genotypes.

Two complementary mathematical methodologies were used to query the raw RNA-seq data for ADPKD-specific gene expression: (1) statistical analysis of differential gene expression (DGE); and (2) unbiased data simplification by principal component analysis (PCA)^33,34^.

**Figure 3:**
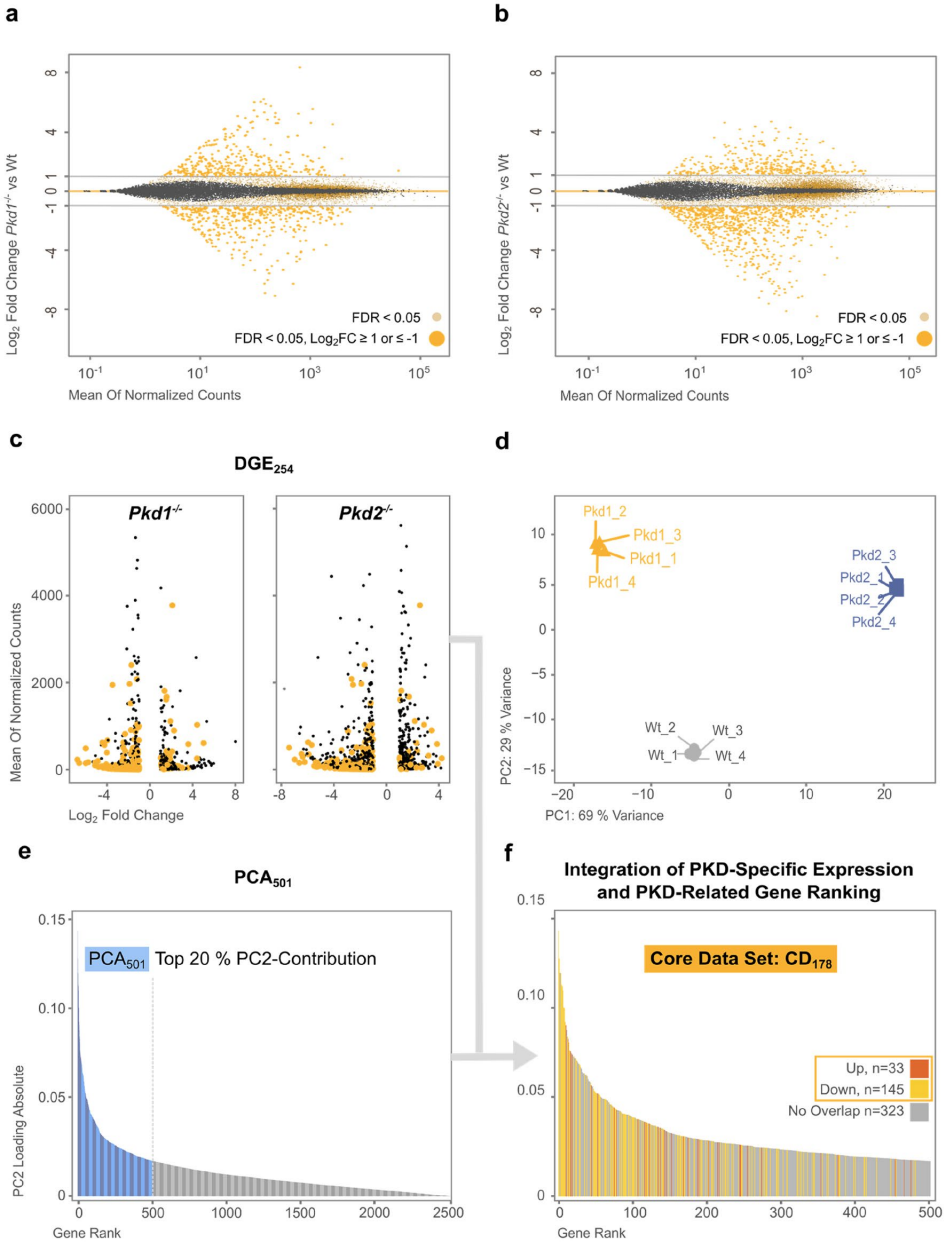
Complementary mathematical analysis of Pkd1- and Pkd2-responsive transcripts identified a core ADPKD data set of 178 consistently regulated genes (CD_178_). To identify genes involved in ADPKD pathogenesis, two independent mathematical approaches were used: (1) statistical analysis of differential gene expression (DGE); and (2) unbiased data simplification by principal component analysis (PCA). (**a**) and (**b**) Applying RNA-seq on Pkd1- and Pkd2-deficient mIMCD3 cells revealed a total of 791 differentially expressed genes in Pkd1^−/−^ and 1250 in Pkd2^−/−^ cells (large, dark-yellow dots: FDR < 0.05, log_2_ fold change ≥|1|). In Pkd1^−/−^ cells, > 54% (429 genes) and in Pkd2^−/−^ cells, > 63% (790 genes) were downregulated. (**c**) Filtering DGE of Pkd1^−/−^ and Pkd2^−/−^ for concordant changes provided 254 genes (DGE_254_), of which 38 were up- and 216 downregulated genes (orange dots). (**d**) 10% most variant genes were input for PCA (n = 2,506, with reads obtained for 25,062 genes). Principal component 1 (PC1) mainly accounted for non-Pkd-related differences between wildtype, Pkd1 and Pkd2 cells. PC2 discriminated Pkd1^−/−^ and Pkd2^−/−^ from wildtype, accounting for 29% of total variance. (**e**) Since PC2 correlated with Pkd1- and Pkd2-deficiency, genes were ranked according to their PC2 contribution, and the top 20% (n = 501, marked in blue) were selected for further data analysis (PCA_501_). (**f**) Comparison of DGE_254_ and PCA_501_ identified 178 candidate genes specifically responding to Pkd1- and Pkd2-deficiency in mIMCD3 cells (core data set; CD_178_).

For DGE, statistical comparison of wild-type, *Pkd1*^−/−^ and *Pkd2*^−/−^ cells identified significant differences in gene expression (Fig. 3a,b). Given that the likelihood of statistically significant results to reflect a true effect is dependent on the respective effect size, we defined DGE positive results by false discovery rate (FDR) < 0.05, and log_2_ fold change ≥|1|^35,36^. By this definition, 781 genes in *Pkd1*^−/−^ and 1150 in *Pkd2*^−/−^ were identified as differentially expressed vs. wildtype (Fig. 3a,b, Tables S1, S2). From these, the DGE-based Pkd-specific renal tubular gene expression profile was compiled by filtering for concordant changes. Hence, DGE identified 254 (38 up- and 216 down-regulated; DGE_254_) Pkd-specific genes (Fig. 3c and Table S3).

For PCA, raw RNA-seq data was input to unsupervised eigenvector-based multivariate analyses. This dimensionality reduction confirmed the close correlation of individual genotypes (Fig. 3d). Two principal components (PC) explained most sample variance, PC1 with 69% and PC2 with 29%. The biological meaning of this data structure may be inferred by the two key factors differentiating the evaluated mIMCD3 cell lines: (1) random clonal variation; and (2) Pkd-dependent gene expression. Because PC2 correlated with *Pkd1*- and *Pkd2*-deficiency, genes were ranked by PC2 weighting and the top 20% (n = 501; PCA_501_) were selected for comparison with the DGE_254_ data set (Fig. 3e and Table S4).

Comparison of the independent DGE_254_ and PCA_501_ data sets identified 178 distinct genes specifically and concordantly responding to *Pkd1*- and *Pkd2*-deficiency in mIMCD3 cells (core data set; CD_178_) (Figs. 3f, S3 and Table S5). Crucially, the integration of *Pkd1* and *Pkd2* signatures should strongly focus CD_178_ on transcripts relevant for ADPKD pathogenesis.

### Function-based clustering of the CD_178_ core ADPKD data set isolated key pathways mediating cyst pathophysiology

Conceptually, CD_178_ provided an unbiased look at the transcriptional activity of Pkd-deficient kidney cells. To uncover the cellular signaling pathways represented by these core ADPKD genes, we next linked prior biological knowledge to the newly generated CD_178_ expression data. Analyses were performed on protein and transcript levels.

**Figure 4:**
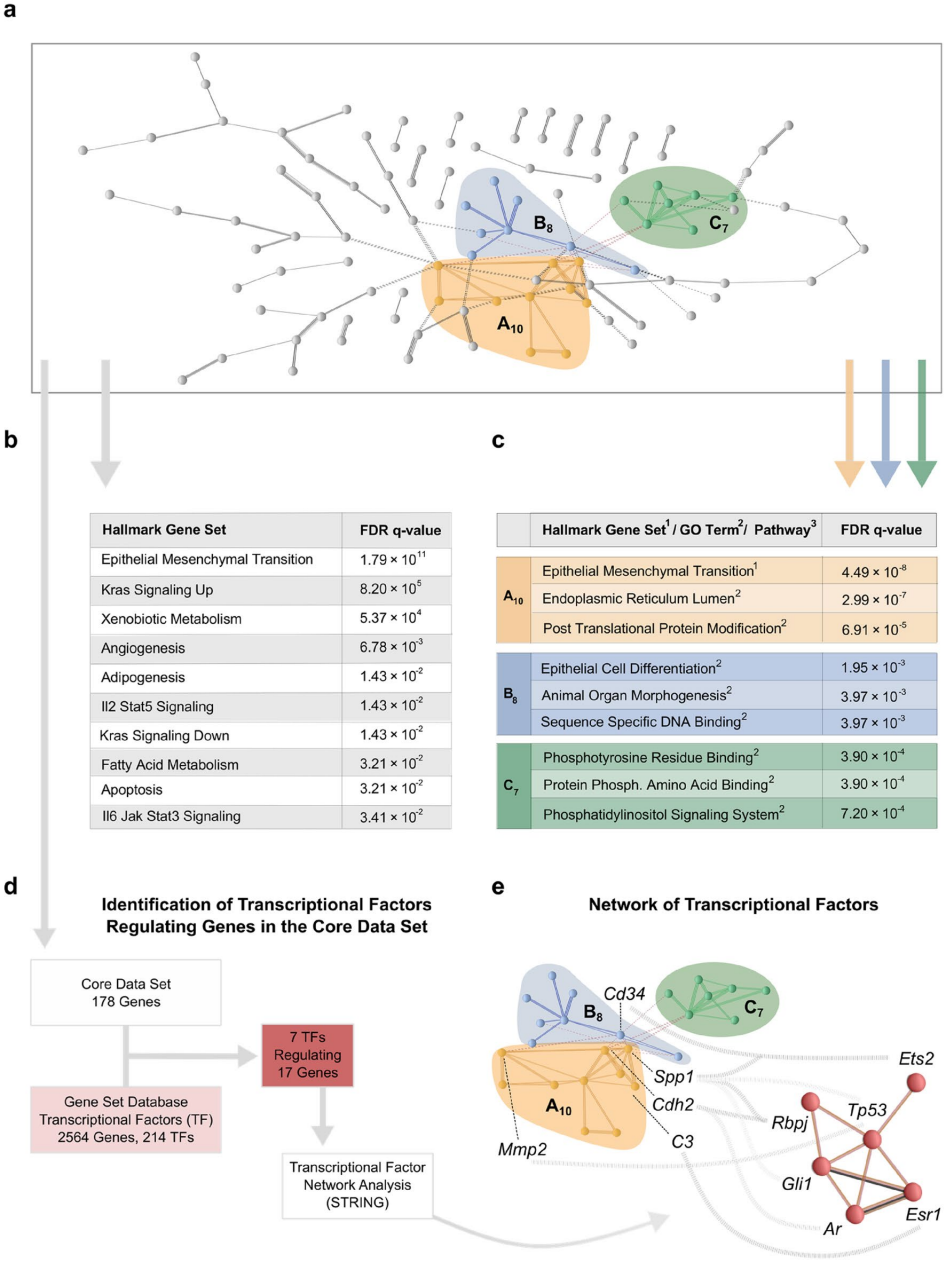
Function-based mapping of CD_178_ identified ADPKD-related protein networks. (**a**) CD_178_ was used as input for STRING network analysis^37^. No annotations were available for 2610203C20Rik and 6330403L08Rik. Application of the k-means algorithm to CD_178_ revealed 3 distinct clusters: A with 10 proteins (A_10_); B with 8 proteins (B_8_); and C with 7 proteins (C_7_; total = ABC_25_). (**b**) and (**c**) to facilitate interpretation of the observed clusters, gene set enrichment analysis (GSEA) was applied to both CD_178_ and ABC_25_ (FDR < 0.05). ABC_25_ genes were subjected to an extended analysis, comprising hallmark gene sets, gene ontology (GO) analysis, transcription factor target analysis and evaluation of the Kyoto Encyclopedia of Genes and Genomes (KEGG)^42,96–98^. See Table S7 for full results. (**d**) To elucidate CD_178_ transcriptional regulation, ShinyGO and GeneSetDB meta-databases were queried (FDR < 0.05)^41,42^. This revealed a total of 17 CD_178_ genes regulated by 7 trancriptional factors (TF_7_). (**e**) 6 of 7 TF_7_ were shown to form a k-means based cluster. Also, ABC_25_ identified several regulatory cascades: 5 ABC_25_ genes (Cd34, Spp1, Cdh2, C3 and Mmp2) were linked to 6 of the TF_7_ (Ar, Esr1, Ets, Gli1, Rbpj, Tp53). Spp1 and Cdh2 were regulated by more than one TF_7_, whereas Ets2, Rbpj and Tp53 regulated more than one ABC_25_ gene.

Protein–protein associations within CD_178_ were queried using the STRING platform^37^. 176 CD_178_ genes matched with proteins in the STRING database (Fig. 4a). Notably, STRING network analysis separated 3 highly connected k-Means clusters within CD_178_: (A) with 10 proteins (A_10_); (B) with 8 proteins (B_8_); and (C) with 7 proteins (C_7_; total = ABC_25_) (Fig. 4a). To interpret the identified protein interactions we performed unguided as well as cluster-guided gene set enrichment analysis (GSEA) using a FDR < 0.05^38^.

Unguided GSEA correlated previously defined hallmark gene sets to CD_178_; with *Epithelial Mesenchymal Transition* (EMT) being the most significantly correlated hallmark gene set (FDR = 1.79 × 10^–11^), supporting the previously described role of EMT in ADPKD cystogenesis (Fig. 4b)^16,39^. Given the smaller size of ABC_25_ and, hence, smaller possibility space, cluster-guided GSEA was complemented with gene ontology (GO) analysis, transcription factor target analysis and evaluation of the Kyoto Encyclopedia of Genes and Genomes (KEGG) (Fig. 4c)^40–42^. Interestingly, cluster A_10_ correlated mainly with EMT; and also with factors related to the *Endoplasmic Reticulum Lumen* as well as *Post Translational Protein Modification*. Cluster B_8_ instead comprised genes related to *Epithelial Cell Differentiation*, *Animal Organ Morphogenesis* and *Sequence Specific DNA Binding*, suggesting a role in tissue formation. And cluster C_7_ seemed exclusively related to phosphorylation-dependent cellular signaling (Fig. 4c). For full GSEA results, see Table S6.

The transcriptional regulation of CD_178_ was probed by transcription factor enrichment analysis using the ShinyGO and GeneSetDB meta-databases^41,42^. Seven transcription factors were identified to be significantly enriched in the regulation of CD_178_ (FDR < 0.05; *Ar*, *Cebpd*, *Esr1*, *Ets2*, *Gli1*, *Tp53* and *Rbpj*; TF_7_), controlling at least 17 of the 178 genes in CD_178_ (Fig. 4d,e). Targeted expression analysis of TF_7_ demonstrated for *Esr1* a significant down-regulation in *Pkd1*^−/−^ and *Pkd2*^−/−^ cells (log_2_ fold change = –1.04, FDR = 0.003 and log_2_ fold change = − 1.54 , FDR = 1.78 × 10^–6^, respectively); none of the other 6 TF_7_ factors showed consistent, *Pkd1-* and *Pkd2*-dependent differences in gene expression (Table S7). Because the PC-1/TRPP2 receptor-ion channel structure makes a direct control of cellular transcription unlikely, it is intriguing to speculate that non-transcriptional polycystin signaling regulates TF_7_ activity, which in turn may control PKD-dependent gene expression in kidney tubular epithelial cells^43^. Ca^2+^-regulation and phosphorylation-dependence, for example, have been described for the zinc-finger protein GLI1 and the cellular tumor antigen p53^44,45^. TF_7_ transcription factors may, hence, provide a mechanistic link from PC-1/TRPP2 channel activity to down-stream regulation of cellular gene expression.

**Figure 5:**
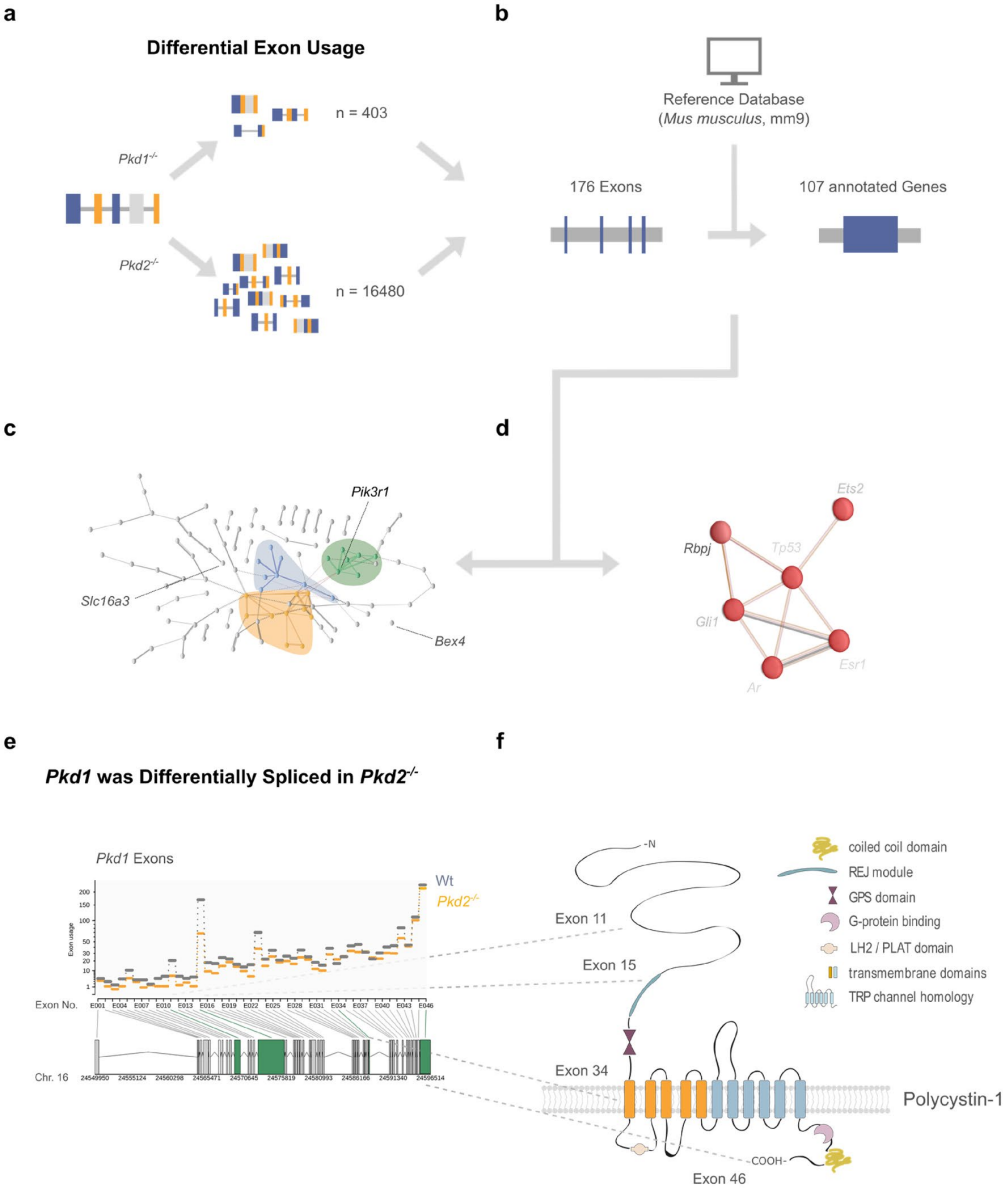
Loss of *Pkd1* and *Pkd2* caused significant alteration in exon usage, including differential splicing of *Pkd1*. (**a**) Differential exon usage in Pkd1^−/−^ and Pkd2^−/−^ cells was assessed using DEXSeq^99,100^. Surprisingly, the number of transcripts with differential exon usage compared to wildtype was > 70-fold higher in Pkd2-deficient cells than in Pkd1^−/−^ cells (n = 16,480 vs. n = 403). (**b**) 176 differentially used exons overlapped between both genotypes. Identified exons mapped to 107 annotated genes and 39 genomic loci without annotation. 107 characterized genes are thus differentially spliced in Pkd1^−/−^ and Pkd2^−/−^ cells. (**c**) Within the CD_178_ core set of genes with Pkd-dependent mRNA expression, Pik3r1, Bex4 and Slc16a3 showed additional Pkd-dependent alterations in exon usage. Interestingly, Pik3r1 was associated with protein phosphorylation in functional cluster C_7_ (Fig. 4c). (**d**) TF_7_ transcription factor Rbpj showed differential splicing in Pkd1^−/−^ and Pkd2^−/−^ cells. Rbpj activity had been linked to the expression of 4 CD_178_ genes: Cdh2, Glul, Mt2 and Spp1 (Fig. 4e). (**e**) and (**f**) further corroborated the functional interdependence of PKD1 and PKD2, Pkd1 exons 11, 15, 34 and 46 showed reduced usage in Pkd2^−/−^ cells. 4 domains of Polycystin-1 (PC-1) were affected: exons 11 and 15 project to the long extracellular N-terminus of PC-1, with exon 15 being part of the receptor of egg jelly module; exon 34 contributes to formation of the transmembrane domains; and exon 46 forms part of the intracellular C-terminus.

Another important contributor to both protein–protein associations and control of gene expression levels is alternative splicing of pre-mRNAs^46^. To date, alternative splicing has not been reported in ADPKD. Analysis of differential exon usage in our wild-type, *Pkd1*^−/−^ and *Pkd2*^−/−^ RNA-seq data identified 176 Pkd-responsive exons in 107 distinct genes (Fig. 5a,b). Within CD_178_, three genes (*Pik3r1*, *Bex4* and *Slc16a3*) showed significant differences in exon usage (Fig. 5c). Similarly, TF_7_ *Rbpj* exon usage seemed to be Pkd-dependent (Fig. 5d). Notably, *Pkd1* exons 11, 15, 34 and 46 were differentially spliced in *Pkd2*-deficient cells (Fig. 5e,f). This indicated that functional TRPP2 is required for both, the amount and the splice variant of *Pkd1* gene expression (Figures S1c, 5e,f). Although direct derivation of biologically meaningful insights from these data is challenging, it reinforced the intimate mutual regulation of PKD genes and PC-1/TRPP2 proteins.

Furthermore, integration of protein association- and transcription factor-derived networks delineated putative Pkd-dependent regulatory cascades in mIMCD3 cells: 5 ABC_25_ genes (*Cd34*, *Spp1*, *Cdh2*, *C3* and *Mmp2*) were linked to 6 of the TF_7_ transcription factors, thus, further connecting phenotypic ADPKD pathogenesis to the unknown cellular functions of *PKD1* and *PKD2* (Fig. 4e).

### Combining published and newly generated data on PKD1 and PKD2 deficiency generated a cross-species transcriptional profile of ADPKD and identified 9 novel targets for future development

The genetic contrast of wild-type and specifically engineered mutant renal tubular epithelial cells provided the opportunity to assess the intricacies of Pkd-dependent transcriptional regulation in simplified cellular isolation. To weigh the relative importance of newly identified CD_178_ pathways in the complex ADPKD in vivo pathophysiology, we next expanded our investigation to the in vitro in vivo correlation of CD_178_-derived pathways.

Renal expression of CD_178_ genes was analyzed by cell type^47^. As expected by the mIMCD3 data source, all CD_178_ genes were consistently expressed along the nephron without particular cortical or juxtamedullary enrichment (Figure S4a–c). Within the nephron, parietal cells (> 60%), collecting duct (> 40%) and medullary pelvis epithelium (> 50%) show the highest expression of CD_178_ genes (Figure S4a–c).

To evaluate the relevance of the newly identified CD_178_ genes for ADPKD pathophysiology we performed a comprehensive cross-species meta-analysis of PKD-dependent RNA expression. At the time of analysis, data were available from six studies evaluating *PKD1*-dependent transcription and one evaluating loss of *Pkd2*: one in renal tubular cells; five in mutant mouse renal tissue; and one in cystic human kidneys (Table S8)^12–16,18^. Data from non-orthologous models of ADPKD were not included in the analysis. For internal validation, we furthermore performed RNA-seq of whole kidneys from littermate control and adult-onset conditional *Pkd1*^−/−^ mice (n = 7; *Pkd1*^*tm2Ggg*^/*Pax8*^*rtTA*^; induction from post-natal day 28 to 42; RNA isolation in week 12) (Figure S5a–c).

**Figure 6:**
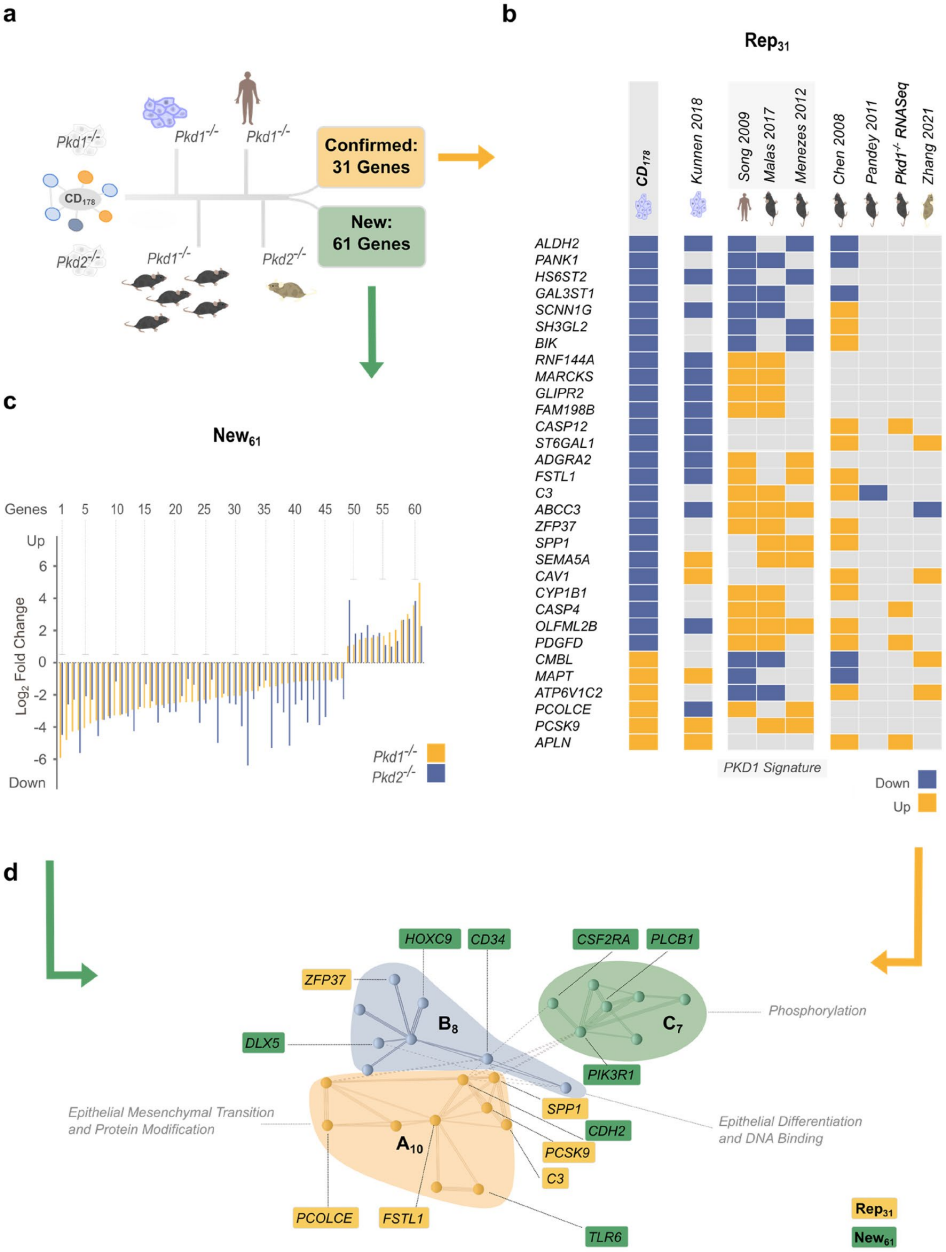
Cross-species meta-analysis of independent ADPKD transcriptomic profiles. (**a**) 7 independent Pkd1^−/−^ transcriptomic studies were chosen for a meta-analysis approach, comprising cell culture, mouse tissue as well as human kidney samples^12–16,18^. 6 studies had been previously published, 3 in another meta-analysis^15^. 1 mouse model (Pkd1^−/−^ RNA-seq) was established for independent confirmation (Figure S5). 31 genes were identified in the core data set and at least 3 other studies. 61 genes are reported in the core data set only. (**b**) Of 31 confirmed genes Rep_31_, 2 showed concordant upregulation, 4 downregulation. Interestingly, 10 genes are concordantly regulated in cell culture models with discordant regulation in animal/human models. (**c**) Complementing published data sets, CD_178_ combined regulation by Pkd1^−/−^ and Pkd2^−/−^. A total of 61 genes appeared in CD_178_ only (New_61_). (**d**) Combining the cross-species meta-analysis with function-based mapping and gene enrichment analysis of ABC_25_ highlighted 8 yet unreported and 6 confirmed genes. Both, confirmed and new genes were represented in clusters A_10_ and B_8._ Interestingly, none of the Rep_31_ but 3 of New_61_ genes were represented in functional cluster C_7_. Thus, combining Pkd1^−/−^ and Pkd2^−/−^ identified new targets for in-depth analysis of ADPKD pathogenesis.

Pooled analysis of aggregated data showed that expression of 117 CD_178_ genes had been described previously as *PKD*-dependent (Figure S6a). Of those, 31 replicated in ≥ 3 independent transcriptional profiles (Rep_31_), with six genes (*ALDH2*, *APLN*, *GAL3ST1*, *HS6ST2*, *PANK1* and *PCSK9*) showing concordant regulation across studies, substantiating their pathophysiological relevance in ADPKD (Figs. 6a,b, S6b,c). 61 *PKD1/2*-dependent genes in CD_178_, on the other hand, had not been associated with ADPKD before (New_61_) (Fig. 6a,c).

To expand our mechanistic understanding of New_61_ in PC-1/TRPP2 signaling and identify novel targets for development in ADPKD, we probed the pathophysiological relevance of respective genes by applied bayesian inference^48^: (1) Through identification, New_61_ genes were selected to show significant concordant changes (log_2_ fold change ≥|1|) in *Pkd1*- and *Pkd2*-deficient mIMCD3 cells (Fig. 6c). (2) Analysis of renal cell type-specific mRNA profiles and comparison to Rep_31_ expression validated disease relevant tissue expression for 49 New_61_ genes (Figure S4)^49^. And importantly, (3) qualitative Rep_31_-based pathway mapping showed a significant functional correlation of Rep_31_ and New_61_. This cross-correlation was most prominent in the ABC_25_ cluster, which directly linked 6 replicated (*C3*, *FSTL1*, *PCOLCE*, *PCSK9*, *SPP1* and *ZFP37*) and 8 newly identified (*CD34*, *CDH2*, *CSF2RA*, *DLX5*, *HOXC9*, *PIK3R1*, *PLCB1* and *TLR6*) genes (Fig. 6d). It is, hence, obvious to hypothesize about the relative contributions of ABC_25_ genes and pathways in loss-of-PKD-dependent cystogenesis and tissue transformation. *FSTL1*, for example, has been linked to kidney fibrosis and *TLR6* to proinflammatory signaling pathways in tubulointerstitial disease^50,51^.

Taken together, our unbiased, model-guided meta-analysis of PKD-responsive transcription clarified conserved cellular signaling pathways in cystogenesis and identified 8 novel high confidence targets for in-depth analysis in ADPKD.

## Discussion

Transcriptomics has emerged as a powerful systems approach for exploring cellular responses to disease-causing mutations^52^. Some discoveries have already translated to the development of novel preventative and therapeutic strategies, including enzyme replacement therapies in deficiency disorders, antisense oligonucleotide therapies for spinal muscle atrophy and channel modulators for gain-of-function channelopathies^53–55^. However, extracting medically relevant changes in mRNA expression from large transcriptional profiles is a challenging task. The indirect link between mRNA abundance and respective protein activity is a key biological limitation^56^. Experimental setup, choice of model system or stage of disease generally limit validity^57^. The limited concordance of previous ADPKD transcriptomic analysis, particular from animal models clearly illustrate these challenges^15,19^. Here, we applied a genetics-guided data science approach to address these constraints and to reveal core genes and transcriptional pathways that have a high likelihood to promote ADPKD pathology. Methodologically, the kidney epithelium-specific transcriptome of ADPKD was profiled by comparing wild-type mIMCD3 cells with one clonal *Pkd1*^−/−^ and one *Pkd2*^−/−^ mIMCD3 cell line. Unbiased differential gene expression (DGE) and principal component analysis (PCA) were used to minimize the probability of erroneous inference and provide a robust ADPKD reference profile for more complex disease models.

Data simplification by PCA is designed to reduce data dimensions and facilitate approximation of data. Data dimensionality in PCA is mathematically reduced by transforming a number of correlated variables into uncorrelated principal components (PCs). In our experiment, PC1 explained 69% of sample variance and PC2 29%. PC1 is most likely reflective of non-genetic clonal variation due to epigenetics and stochastic gene expression as well as divergent *Pkd1/2* signaling in mIMCD3 cells (Figs. 3d, S3a–e). Furthermore, the contribution of PC1 to total sample variance is in line with previous analyses of clonal variation in various cell types^58,59^. PC2, on the other hand, better correlated with *Pkd1/2* gene expression (Fig. 3d). The significant overlap of differentially expressed genes (DGE_251_) and PC2-based PCA_501_ in the *Pkd1/2*-dependent CD_178_ core data provided critical evidence for this argument (Fig. 3f).

The potential biological relevance of CD_178_ in vivo was subsequently validated by meta-analysis of independent ADPKD transcriptomes, including new data from a *Pkd1*^−/−^ mouse model (Fig. 6a–d). Putative confounding factors of this approach remain our incomplete understanding of the relevant ADPKD phenotype in vitro as well as missing or biased information in reference biobanks^60^. Still, available data was sufficient to identify the *PKD1/2* > TF_7_ > ABC_25_ hierarchical regulatory network, which may comprise core pathways amplifying transient polycystin-dependent ion flux into stable cellular differentiation and renal tubular structure (Figs. 4e, 6d). Derived from the largest volume of ADPKD mRNA expression data available, the *PKD1/2* > TF_7_ > ABC_25_ signaling cascade—and in particular the 6 replicated and 8 newly identified genes—generated novel, testable hypotheses that may broaden our understanding of PKD-dependent tissue homeostasis in health and disease.

For example, sex has been described as significant determinant of ADPKD disease severity^61^. Men with ADPKD often have worse renal cystic disease but less severe liver cystic disease^62^. Supported by the male origin of mIMCD3 cells, *PKD1/2* > *AR* > *CDH2* signaling in cluster A_10_ may contribute to this phenotype (Figs. 4e, 6d and Table S9). The *AR* gene in TF_7_ encodes the steroid-hormone activated androgen receptor, which regulates eukaryotic gene expression and affects cellular proliferation and differentiation in many target tissues^63^. Consistent with a putative input from PC-1/TRPP2, AR transcription factor activity is modulated by bound coactivator and corepressor proteins as well as post-translational modifications^64^. AR-regulated *CDH2* encodes cadherin-2, a Ca^2+^-dependent cell adhesion protein that is known to be important for the establishment and maintenance of the multicellular structure of organs^65^. Notably, renal proximal tubular cells are the only epithelial cells in the adult organism expressing cadherin-2 as major cell–cell adhesion protein^66^. Up-regulation of *CDH2* is a hallmark of EMT, which has been described extensively for cyst-lining cells (Figure S6d)^67,68^. Further investigation of *PKD1/2* > *AR* > *CDH2* may hence link several important disease-modulating factors in men with ADPKD.

Within the *Epithelial Cell Differentiation* cluster B_8_, *PKD1/2* > *ETS2* > *CD34* may provide another interesting model to test (Figs. 4e, 6d). *ETS2* encodes the epithelium-specific, winged helix-turn-helix E26 transformation-specific proto-oncogene 2 transcription factor (ETS2) that regulates diverse cellular activities, including apoptosis, cell growth, adhesion, the extracellular matrix and other transcription factors^69^. ETS2 is a labile protein with a short half-life^70^. ETS2 transcription factor activity is regulated by E3 ligase-dependent ubiquitination and subsequent proteasomal degradation^71,72^. Interestingly, PC-1/TRPP2 has already been shown to regulate the activity of two E3 ligases, SIAH-1 and c-CBL^73–75^. Also, ETS2 is a downstream effector of the RAS/RAF/ERK pathway that promotes disease pathogenesis in ADPKD^76^. CD34 is regulated by ETS2 and is a common marker for diverse progenitor cells with progressive downregulation on more mature cells^77^. Functionally, CD34 is a sialomucin-type single-transmembrane glycophosphoprotein closely related to podocalyxin that seems to alter cellular adhesive properties^78,79^. CD34 is furthermore intracellularly coupled to CRKL, which has been associated with congenital kidney anomalies in DiGeorge Syndrome, including microcystic tubules and glomeruli^80,81^. The observed PKD-dependent over-expression of *CD34* may therefore contribute to cystogenesis by causing cellular de-differentiation that interferes with renal tubular stability (Figure S6d).

In cluster C_7_ we identified *CSF2RA*, *PIK3R1* and *PLCB1* as novel genes responsive to *PKD1* and *PKD2*, but not enough biobank information was available to automatically develop transcription-based regulatory signaling models (Figs. 4e, 6d). However, functional links to ADPKD have been described previously for *PIK3R1* and *PLCB1*. *PIK3R1* encodes phosphoinositide-3-kinase regulatory subunit 1 (PIK3R1), the major suppressive regulatory subunit of phosphoinositide 3-kinase (PI3K). PI3K participates in a broad range of regulatory processes, including cell growth, proliferation, metabolism and secretion^82^. In cancer, loss of PIK3R1 has been shown to promote EMT and cellular proliferation by over-activation of downstream AKT signaling^83^. In ADPKD, increased PI3K/AKT signaling has been described as disease modifying, but both, loss and gain of *PKD1* have been linked to mis-regulated pathway activity^84–88^. Increased PIK3R1 protein expression has been observed in conditionally immortalized human tubular epithelial cells from ADPKD patients^89^. Disease status, species differences or the observed Pkd-dependent *Pik3r1* exon usage may reconcile these divergent findings, but additional experimental data are needed to clarify the molecular function of PIK3R1 in ADPKD. *PLCB1* encodes phospholipase C beta 1 (PLCβ1), which catalyzes the formation of inositol 1,4,5-trisphosphate (PiP_3_) and diacylglycerol (DAG) from phosphatidylinositol 4,5-bisphosphate (PiP_2_). PLCβ1 uses Ca^2+^ as a cofactor and plays an important role in the intracellular transduction of many extracellular signals^90^. Gα_q_-mediated activation of PLC has been described downstream of PC-1^91^.

Our transcriptomic analyses yielded novel, testable hypotheses and highlighted our still rather limited understanding of cellular physiology. Publicly available biobank data allowed for the identification and cross-correlation of ABC_25_, but were insufficient to develop unbiased hypotheses for most variations in PKD-dependent gene expression. To date, combination of sophisticated model systems and high-resolution methods yield volumes of relevant transcriptomic data. Yet, interpretation has proven challenging when it comes to deriving biological meaning, as most types of systemic analysis, such as over-representation analysis, GSEA and signaling pathway impact analysis, rely to some degree on a priori knowledge of the pathways, the biological role, or the molecular function of genes^38,92^. As such, annotations have been instrumental for understanding molecular signatures of many diseases. But annotation-based approaches have significant drawbacks that limit the insights that can be gleaned from respective analyses. For instance, there is a strong underlying assumption that orthologous genes share similar biological functions. This may overemphasize highly conserved cellular processes and potentially overlook important species-specific and/or tissue-specific functions^93^. Similar network effects cause well-studied genes to continuously accrue data, while the non-randomness of missing annotations is more likely to disadvantage primate specific genes or new targets without a wealth of literature^94,95^. With more data becoming publicly available, future in silico re-analyses of CD_178_ have therefore the intriguing potential to further clarify core functional connections currently still hidden in the ADPKD transcriptomic profile.

Taken together, our data indicate the robust power of integrative transcriptomics to select targets for subsequent functional studies in ADPKD, including the 6 replicated (*C3*, *FSTL1*, *PCOLCE*, *PCSK9*, *SPP1* and *ZFP37*) and 8 newly identified (*CD34*, *CDH2*, *CSF2RA*, *DLX5*, *HOXC9*, *PIK3R1*, *PLCB1* and *TLR6*) genes (Fig. 6d).

## Methods

### Contact for reagent and resource sharing

Further information and requests for resources should be directed to the Lead Contacts, Michael Köttgen (michael.koettgen@uniklinik-freiburg.de) and Alexis Hofherr (alexis.hofherr@uniklinik-freiburg.de).

### Key resources

Details on key resources are listed in the supplementary information Key Resources Table.

### Experimental model details

#### Cell culture

Murine inner-medullary collecting duct cells (mIMCD3) were isolated from healthy mice expressing the early region of simian virus SV40 (Tg(SV40E)Bri/7) to sustain continuous proliferation in vitro^22^. Wild-type mIMCD3 cells are diploid, consistently differentiate and replicate collecting duct-like transport activity^22–25^. *Pkd1*^−/−^ and *Pkd2*^−/−^ mIMCD3 cells have been described previously^28^. Independent deletion of *Pkd1* (CRISPR) and *Pkd2* (TALEN) was confirmed by DNA sequencing and validated by RNA-Seq: mIMCD3 *Pkd1*^−/−^ (Δ-chr. 17: 24,550,055–24,594,963/Δ-chr. 17: 24,550,106–24,594,903); mIMCD3 *Pkd2*^−/−^ (Δ-chr. 5: 104,478,281–104,490,878)^28^. RNA-seq read mapping and de novo transcriptome assembly did not indicate significant genome-editing induced off-target effects in *Pkd1/2* cells. Flow cytometry-based DNA content measurement showed normal ploidy in all cell lines. Loss of respective PC-1 and TRPP2 expression was demonstrated by Western blot. Overall viability, proliferation and differentiation, including ciliation, basal lamina formation and transepithelial electrical resistance, were similar in wild-type and mutant cells^28^.

Cells were cultured in a mix of Dulbecco’s modified eagle’s and F12 medium (DMEM-F12; Lonza), which was supplemented with 10% fetal bovine serum and 1% Penicillin–Streptomycin (10,000 U Penicillin and 10 mg / ml Streptomycin; both Sigma Aldrich). Cells were grown at 37 °C and 10% CO_2_. PCR-based testing for mycoplasma contamination was performed regularly. New ampules were started after ~ 25 passages. Cells were harvested using 0.25% Trypsin–EDTA (Gibco). Cell counts were performed by Countess II cell counter (Thermo Fisher).

### Method details

#### Protein isolation, SDS-PAGE, Western blot and ECL detection

Cells were grown to epithelial confluency (5 days). Cells were lysed, proteins were isolated, processed in SDS-PAGE and Western blot and detected by electrochemiluminescence as described previously^101^. For TRPP2-immunoprecipitation, mouse anti-TRPP2 antibody was used (Santa Cruz Biotechnologie Inc., Dallas, USA). Beta-actin was targeted using anti-beta-actin antibody (Sigma Aldrich, St. Louis, USA). Luminescence was detected by a 16-bit ChemoCam system (Intas).

#### Immunofluorescence

Indirect immunofluorescence staining of cells has been described previously^102^. Briefly, after reaching full confluency cells were permeabilized using Triton X-100 (Carl Roth GmbH, Karlsruhe, Germany) and fixed by paraformaldehyde (Electron Microscopy Sciences, Hatfield, PA, USA). Cells were stained by primary (1:50 anti-ZO1 rat, Santa Cruz Biotechnology, Dallas, USA, 1:800; anti-alphaTubulin rabbit, Sigma Aldrich, St. Louis, USA) and secondary antibodies (1:1000 Anti-rat IgG, Cy3, Jackson Lab. Inc., Waltham, USA; anti-rabbit IgG, Cy3; Jackson Lab. Inc., Waltham, USA) in PBS. DAPI (Sigma-Aldrich) was added in a dilution of 1:1000 to visualize DNA. Bright-field images were recorded using an Axio Vert Observer microscope Z1 (Zeiss).

#### RNA isolation

For wildtype/Pkd comparison, six 10 cm cell culture dishes were grown per genotype to full confluency and ciliation, starting with 5 × 10^6^ cells per dish. RNA Isolation was performed 4 days after full confluency according to manufacturer’s protocol using the RNeasy Plus Mini Kit (Qiagen), harvesting 2 × 10^6^ cells per dish. Purification of RNA samples was performed by solvent extraction with 100% ethanol followed by precipitation with ammonium acetate.10 µl glycogen was added to 100 µl RNA solution. RNA was solved in 10 µl ammonium acetate (7.5 M) and 250 µl ethanol (100%). After 30 min at − 80 °C RNA was precipitated and separated by centrifugation for 30 min with 13,000 rpm at 4 °C. RNA was then washed twice in 200 µl cold ethanol (80%) followed by 30 min centrifugation with 13,000 rpm at 4 °C. RNA pellets were dried for 5 min at room temperature and resuspended in 10–15 µl RNAse-free water. Quality of RNA was checked by a Nanodrop photometer (Thermo Fisher). Four of the six samples were chosen for RNA-seq according to highest ratios for A260/280 (> 2.0) and A260/230 (> 2.0). RNA-seq was performed by GATC (Konstanz, D) on Illumina HiSEQ2500.

#### RNA sequencing (RNA-seq) analysis

RNA-seq was performed in samples from wild-type as well as from one *Pkd1*^−/−^ and one *Pkd2*^−/−^ mIMCD3 clone. Despite multiple sampling, untargeted analysis of such clonal cell lines may be confounded by undetected, random genome editing-induced off-target effects or clonal artifacts. To minimize false discovery, mIMCD3-based transcriptomic profiling of ADPKD was hence restricted to consistent changes across the two independent Pkd genotypes. Data was analyzed with Galaxy Europe^103^ (https://usegalaxy.eu/). Reads were first trimmed and quality checked with Trim Galore (Galaxy Tool Version 0.4.3.1) and FastQC (Galaxy Tool Version 0.72), respectively. Reads were aligned to the mouse genome version mm10 (GRCm38) applying HISAT2 (Galaxy Tool Version 2.1.0 + galaxy3)^104^. Mapped reads were counted with htseq-count (Galaxy Tool Version 0.9.1)^105^. Differential gene expression (DGE) was tested using DESeq2 (Galaxy Tool Version 2.11.40.4) using a cutoff of log_2_ fold change of ≥ 1 for upregulated and ≤ − 1 for downregulated genes^35,106^. False discovery rate of DGE was controlled with a p value < 0.05 adjusted for multiple testing (Benjamini–Hochberg procedure). Principal Component Analysis (PCA) was performed using DESeq2 in R^106^. Of 25′061 genes with reads obtained in the RNA-seq analysis, 10% most variant genes were selected as input for PCA (n = 2506). Data was visualized using the R packages: pcaExplorer and pheatmap^107^.

#### Alternative splicing analysis

The reads processed in the RNA-seq analysis were also aligned to the reference genome and tailored for StringTie tool with HISAT2 (Galaxy Tool Version 2.1.0 + galaxy3)^104^. De novo transcript reconstruction was conducted with StringTie (Galaxy Tool Version 1.3.6)^108^. The transcriptome was assembled using StringTie-merge (Galaxy Tool Version 1.3.6). Assembled transcripts were compared to the reference annotation (*Mus musculus*, mm9) with GFFCompare (Galaxy Tool Version 0.11.2)^109^. Exon abundancies were then estimated using DEXSeq-Count (Galaxy Tool Version 1.28.1.0)^99,100^. Differential exon usage (DEU) was tested with DEXSeq (Galaxy Tool Version 1.28.1 + galaxy1)^99,100^. False discovery rate of DEU was controlled with a p-value < 0.05 adjusted for multiple testing (Benjamini–Hochberg procedure).

#### CellNet analysis

CellNet Analysis of RNA-seq data was performed as described previously^27^. Data was uploaded to an Amazon web services (AWS) instance. No alterations to CellNet standard settings were performed. For the original code to work with the chosen AWS platform, all AWS-related URLs (https://s3.amazonaws.com/) were changed to https://s3.console.aws.amazon.com/s3/, e.g. in the "cn_s3_fetchFastq" function.

#### Kidney cell explorer analysis

Kidney Cell Explorer Analysis was performed using^47^ https://cello.shinyapps.io/kidneycellexplorer/. "Adult mouse kidney" was selected as reference data set. CD_178_ were used as input by pasting the respective gene IDs in the search field. Expression was displayed in "Average expression (rescaled)" mode and "RdYIBu" Palette. Genes were counted as "expressed" if rescaled average expression was > 0, e.g. if color was lighter than dark blue (HEX code #4575b4ff.).

#### Nephro cell analysis

Nephro cell analysis was performed using http://nephrocell.miktmc.org/. "Adult normal kidney" was selected as reference data set. Rep_31_ and New_61_ were used as input; one gene at a time. Violin plots displayed were analyzed and divided into 5 categories: 0 = not measured in the nephrocell data set; 1 = no expression; 2 = low expression, no violin plots displayed; 3 = high expression, violin plots displayed; 4 = very high expression, striking violin plots displayed. A heatmap was generated to visualize these values.

#### Transcription factor enrichment analysis

Testing for transcription factors regulating the core data set genes was performed with ShinyGO (Tool Version 0.51) using the function TF.Target.TFacts^41,42^. False discovery rate was set to < 0.05.

#### Pathway analysis

Functional protein network and transcriptional factor network were determined using the STRING (Search tool for the retrieval of interacting genes/proteins) database version 11.0^37^ (https://version-11-0.string-db.org/).

Functional enrichment analysis was performed against the Molecular Signature Database (MSigDB) v7.1 using standard hypergeometric distribution with correction for multiple hypotheses testing using FDR < 0.05 according to Benjamini and Hochberg^38,42^. Pathway databases used: Hallmark Gene Sets, Positional Gene Sets, GO Gene Sets, KEGG, All Transcription Factor Targets.

#### Cross-species meta-analysis

Available *PKD1*-responsive transcriptional data was retrieved from a literature research covering publications between 2008 and 2018^12–17^. Transgenic models were not considered for comparison. Studies were included if they provided lists of differentially expressed genes and raw data had been deposited for public access. No *PKD2* data matching above criteria were found. Ref.^18^ was included for *PKD2*-responsive expression. Lists of differentially expressed genes were obtained from the primary or supplementary data of the respective publications. Analysis of^13,16^ was based on the previously described *PKD1 Signature* by^15^. Criteria for differential gene expression were adopted from the respective studies.

#### RNA-seq and profiling of Pkd1^tm2Ggg^/Pax8^rtTA^ mice

Pkd1^*tm2Ggg*^/*Pax8*^*rtTA*^ (C57BL/6 genetic background) mice have been described previously^110^. All animal experiments were conducted according to the guidelines of the National Institutes of Health Guide for the Care and Use of Laboratory Animals, as well as the German law for the welfare of animals, and were approved by regional authorities (Regierungspräsidium Freiburg G‐13/18, G‐15/58, and G‐16/28). Mice were housed in a specific pathogen‐free facility, fed ad libitum, and housed at constant ambient temperature in a 12‐hour day/night cycle. Breeding and genotyping were done according to standard procedures. *Pkd1* deletion was induced from post-natal day 28–42. Mice received doxycycline hydrochloride (Fagron) via the drinking water (2 mg/mL with 5% sucrose, protected from light) to induce *Pkd1* deletion. Littermates (lacking either TetO-Cre or Pax8^rtTA^) were used as controls. 2 male control animals and 5 male *Pkd1*^−/−^ animals were sacrificed at a median age of 11.8 weeks. RNA was extracted from kidneys with RNeasy Plus Mini Kit (Qiagen) according to the manufacturer's protocol as a one-step elution with 50 µl RNAse free water per sample. Quantity and quality of RNA was checked by NanoDrop One spectrophotometer (Thermofisher). RNA-seq was performed by GATC Biotech AG (Konstanz, D) on Illumina HiSeq Genome Sequencer. Data analysis was performed using the same work-flow as for CD_178_ (see "RNA Sequencing analysis"). Differentially expressed genes (FDR < 0.05) were overlapped to top 20% genes contributing to principal component 2 in the principal component analysis. Overlapping genes were summarized in the *Pkd1*^−/−^ mouse data set.

### Quantification and statistical analysis

Statistical analyses of RNA-seq data were carried out in Galaxy and R using DESeq2^103,106^. False discovery rate of DGE was set to < 0.05 (Benjamini–Hochberg procedure). The number of biological replicates (n), statistical test performed and resulting significance for each experiment is noted in the figure legend or the corresponding results section of this document. Where applicable, original data for plots is available in supplementary tables. Additional data is available upon request.

## Supplementary Information

Supplementary Information 1.

Supplementary Information 2.

Supplementary Information 3.

Supplementary Information 4.

Supplementary Information 5.

Supplementary Information 6.

Supplementary Information 7.

Supplementary Information 8.

Supplementary Information 9.

Supplementary Information 10.

Supplementary Information 11.

## Data Availability

The raw and processed data from our analyses of RNA-seq will be available in the gene expression omnibus database under the accession number: GSE179947.
