# Supplementary material for: Identification of pathological transcription in autosomal dominant polycystic kidney disease epithelia
Source: Sci Rep. 2021 Jul 23;11:15139. doi: 10.1038/s41598-021-94442-8 (PMC8302622; doi:10.1038/s41598-021-94442-8)
Supplement: Supplementary file 1 — Supplementary Information 1. [file 41598_2021_94442_MOESM1_ESM.docx]

**Supplementary Information**

Identification of Pathological Transcription in Autosomal Dominant Polycystic Kidney Disease Epithelia

Sebastian Friedrich^a,b,¶^, Hannah Müller^a,¶^, Caroline Riesterer^a,¶^, Hannah Schüller^a^, Katja Friedrich^a^, Carlotta Leonie Wörner^a^, Tilman Busch^a^, Amandine Viau^c^, E. Wolfgang Kuehn^a,d^, Michael Köttgen^a,d,‡^, Alexis Hofherr^a,‡^

^a^ Renal Division, Department of Medicine, Medical Center, Faculty of Medicine, University of Freiburg, Hugstetterstrasse 55, 79106 Freiburg, Germany

^b^ Current Address: Department of General Pediatrics, Adolescent Medicine and Neonatology, Center for Pediatrics, Medical Center, Faculty of Medicine, University of Freiburg, Mathildenstrasse 1, 79106 Freiburg, Germany

^c^ Université de Paris, Imagine Institute, Laboratory of Hereditary Kidney Diseases, INSERM UMR 1163, F-75015, Paris, France

^d^ CIBSS – Centre for Integrative Biological Signalling Studies, Freiburg, Germany

^¶^ **These authors contributed equally**

**^‡^ Corresponding authors**

alexis.hofherr@uniklinik-freiburg.de

michael.koettgen@uniklinik-freiburg.de

# Supplementary Information

## Key Resources Table

## Figures S1 – S6

## Tables S1 – S10

# Key Resources Table

| **REAGENT / RESOURCE** | **SOURCE** | **IDENTIFIER** |
| --- | --- | --- |
| **Antibodies** | | |
| Rat monoclonal anti-ZO1 | Santa Cruz Biotechnology,  Dallas, USA | Cat# sc-33725;  RRID: AB_628459 |
| Rabbit monoclonal anti-alphaTubulin | Sigma Aldrich,  St. Louis, USA | Cat# T6793;  RRID: AB_477585 |
| Cy3-AffiniPure Donkey Anti-Rat IgG | Jackson Lab. Inc.,  Waltham, USA | Cat# 712-165-153;  RRID: AB_2340667 |
| Cy3-AffiniPure Donkey Anti-Rabbit IgG | Jackson Lab. Inc.,  Waltham, USA | Cat# 711-165-152;  RRID: AB_2307443 |
| 4′,6-Diamidin-2-phenylindol (DAPI) | Sigma Aldrich,  St. Louis, USA | Cat# D9542 |
| Mouse monoclonal anti-TRPP2  (clone YCE2) | Santa Cruz Biotechnologie Inc., Dallas, USA | Cat# sc-47734;  RRID: AB_672380 |
| Mouse monoclonal anti-beta-actin  (clone AC-15) | Sigma Aldrich,  St. Louis, USA | Cat# A1978;  RRID: AB_476692 |
| **Chemicals, Peptides and Recombinant Proteins** | | |
| 0,25 % Trypsin-EDTA 1 x | Thermo Fisher Scientific Inc., Waltham, USA |  |
| Paraformaldehyde | Electron Microscopy Sciences, Hatfield, PA, USA | Cat# 30525-89-4 |
| **Critical Commercial Assays** | | |
| RNeasy Plus Mini Kit | Qiagen GmbH, Hilden, D | Cat# 74134 |
| **Deposited data** | | |
| RNA-seq raw data | | GEO: **pending** |
| **Experimental models: Cell Lines** | | |
| Mouse: mIMCD3; wild-type | ATCC | CRL-2123 |
| Mouse: mIMCD3; Pkd1^-/-^  (del Chr. 17: 24,550,055 - 24,594,963 /  del Chr. 17: 24,550,106 - 24,594,903) | ^1^ | |
| Mouse: mIMCD3; Pkd2^-/-^  (del Chr. 5: 104,478,281 - 104,490,878) |  | N/A |
| **Software and Algorithms** | | |
| R v4.0 | https://www.R-project.org | |
| RStudio v1.3 | https://www.rstudio.com/ | |
| Galaxy Project | ^2^ | https://www.usegalaxy.org/ |
| Inkscape | https://www.inkscape.org/ | |
| CellNet | ^3^ | https://doi.org/10.1038/nprot.2017.022 |
| Nephro Cell | ^4^ | https://doi.org/10.1172/jci.insight.133267 |
| Kidney Cell Explorer | ^5^ | https://doi.org/10.1016/j.devcel.2019.10.005 |
| STRING database | ^6^ | RRID: SCR_005223 |
| ImageJ Fiji App | ^7^ | RRID: SCR_002285 |
| DESeq2 | ^8^ | RRID: SCR_015687 |

# Supplementary Figures

## **Figure S1:** Validation of engineered mIMCD3 cells as highly differentiated, kidney-specific in vitro model of ADPKD.

**a)** mIMCD3 cells are highly differentiated renal tubular epithelial cells. Here visualized by staining for primary cilia (arrow mark; αTubulin) and cell borders (Zonula occludens 1 (ZO1). Scale bar: 10 µm. **b)** CellNet analysis of active gene-regulatory networks confirmed renal fidelity of mIMCD3 wild-type (Wt), Pkd1^-/-^ and Pkd2^-/-^ cells ^3^. **c)** TRPP2-deficient cells were generated by deletion of Pkd2 exons 4 – 9 and confirmed biochemically. Despite the genomic deletion, remaining levels of Pkd2-transcripts in Pkd2^-/-^ were similar to Wt (3,986 [SEM = 107] mean normalized counts in Pkd2^-/-^ vs. 3,971 [SEM = 390] Wt). Deletion of Pkd1 was association with a significant reduction in Pkd2 mRNA abundance (3,971.25 [SEM = 390] mean normalized counts in wildtype vs. 2,711 [SEM = 114] in Pkd2^-/-^). **d)** Exon usage in Pkd2 was dependent on genotype. Pkd2-transcript in Pkd2^-/-^ was detected mainly for Exon 15 (normalized counts ranging from 1,646 to 2,999 in Wt, Pkd1^-/-^ and Pkd2^-/-^ cells).

**

**

## **Figure S2:** Global analysis of RNA-seq data confirmed consistently good data quality.

**a)** Of the total 47,642 annotated transcripts in Mus musculus, reads were obtained for 25,061, yielding a sequencing depth of 52.60 %. **b)** 4 replicates were used per genotype (wildtype, Pkd1^-/-^ and Pkd2^-/-^). Reads were evenly distributed between replicates, allowing for all to be included in our analysis. **c)** Unsupervised hierarchical clustering of sample-to-sample-distances revealed close proximity between replicates of the same genotype. **d)** Dispersion estimates plotted over average expression strength per gene showed consistent variance values of genes with similar average expression strength before and after statistical shrinkage. Thus, accurate testing for differential expression was warranted. **e)** For Pkd1^-/-^ vs. wildtype and Pkd2^-/-^ vs. wildtype, most p-values are < 0.05, yielding a high proportion of differentially expressed genes.

**

**

## **Figure S3:** Divergent expression in *Pkd1^-/-^* and *Pkd2^-/-^* cells.

**a)** Divergent transcription may contribute to the non-concordant phenotypes observed for loss of PKD1 and PKD2, including the slower course of ADPKD in patients with PKD2 mutations or the PKD1-independent function of PKD2 in left-right organ placement ^9,10^. Comparing Pkd1^-/-^ and Pkd2^-/-^ cells, 1,429 genes were differentially expressed (FDR < 0.05, log_2_ fold change ≥ |1|). 883 (61.7 %) genes were upregulated and 546 downregulated (38,3 %). However, because of the smaller sample size, the power to detect meaningful differences in the comparison of Pkd1- and Pkd2-deficient cells was smaller than in the comparison to wild-type (n = 8 vs. n = 12 with 4 per genotype). **b)** Venn diagram of Pkd-dependent transcription compared to wild-type in mIMCD3 cells, including DGE_254_ in gray, which is the sum of concordantly regulated genes (FDR < 0.05, log_2_ fold change ≥ |1|) (**Figure 3c)**. **c)** Principal component analysis identified principal component 1 (PC1) as discriminating factor between Pkd1^-/-^ and Pkd2^-/-^ cells (**Figure 3d)**. Thus, genes were ranked by PC1 contribution and top 20 % (n = 501, marked in blue) were selected for further analysis. **d)** Overlapping differentially expressed genes from **a)** and genes contributing to PC1 in **c)** identified 437 genes with divergent expression in Pkd1- and Pkd2-deficient cells. **e)** Gene set enrichment analysis on **d)** identified several divergent signaling pathways. See **Table S10** for full results.

**

**

## **Figure S4:** CD_178_ genes were mainly expressed in the collecting duct and parietal epithelium.

Renal expression of CD_178_ genes was analyzed using the Kidney Cell Explorer ^5^. **a)** In cortical nephrons, 60 – 69 % of CD_178_ genes were expressed in parietal epithelial cells and 40 – 59 % in collecting duct cells. **b)** In juxtamedullary nephrons, equally 60 – 69 % of CD_178_ genes were expressed in parietal epithelial cells and 40 – 59 % in collecting duct cells. Specific juxtamedullary nephron segments comprised only 30 – 39 % of CD_178_. **c)** Nephron segments as defined by ^5^. **d)** Expression profiles in specific renal cells were investigated using the Nephrocell tool and translated into a heatmap visualization according to criteria shown ^4^. Rep_31_ genes (**Figure 6**) were used as input and compared to New_61_ genes. Expression profiles were largely similar, with most genes showing low expression (no violinplots displayed in the Nephrocell tool). Relevant tissue expression was confirmed for 49 of New_61_ genes. Three Rep_31_ genes (Aldh2, Cmbl, Spp1) showed marked expression in proximal tubule cells.

**
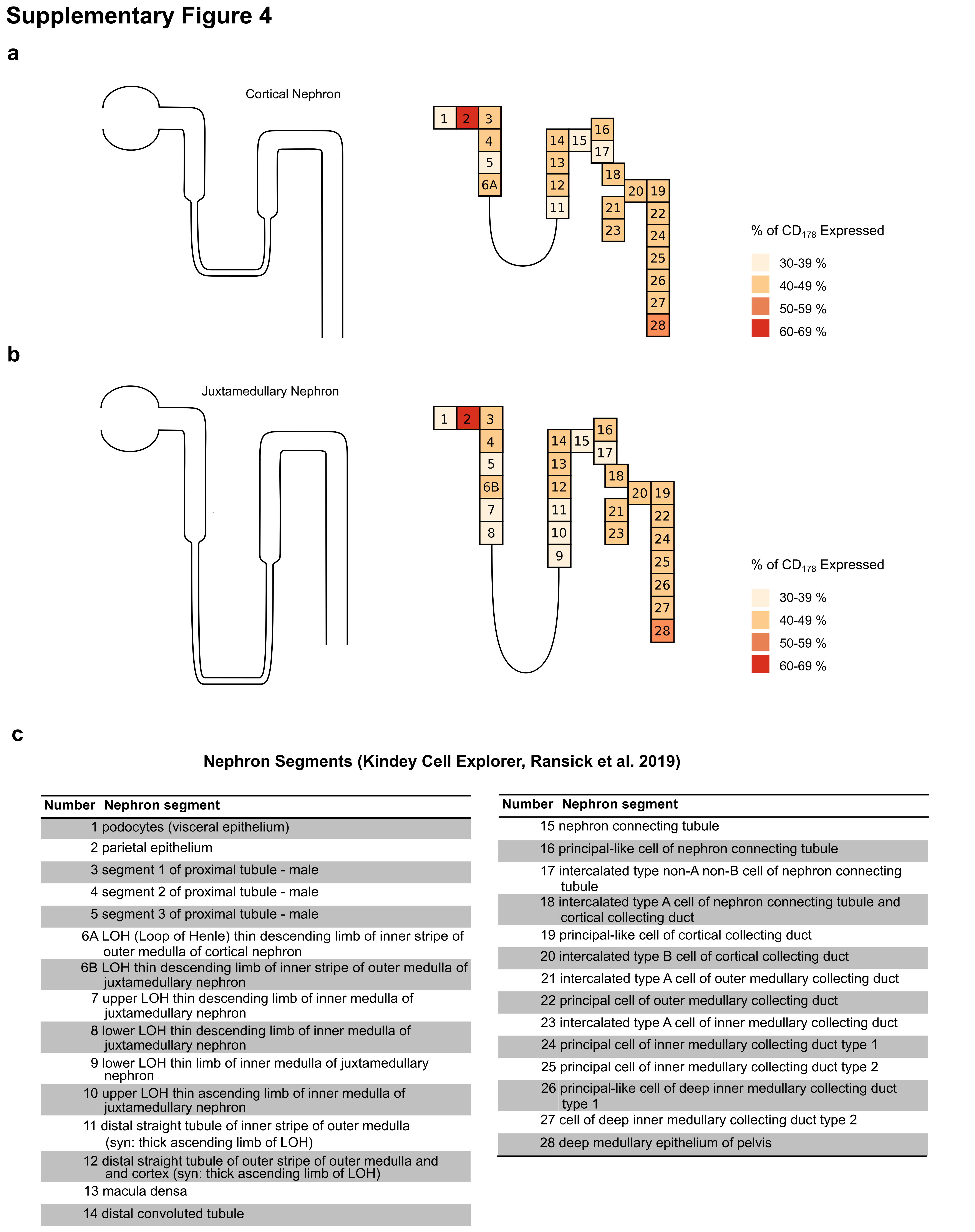
**

**

**

**
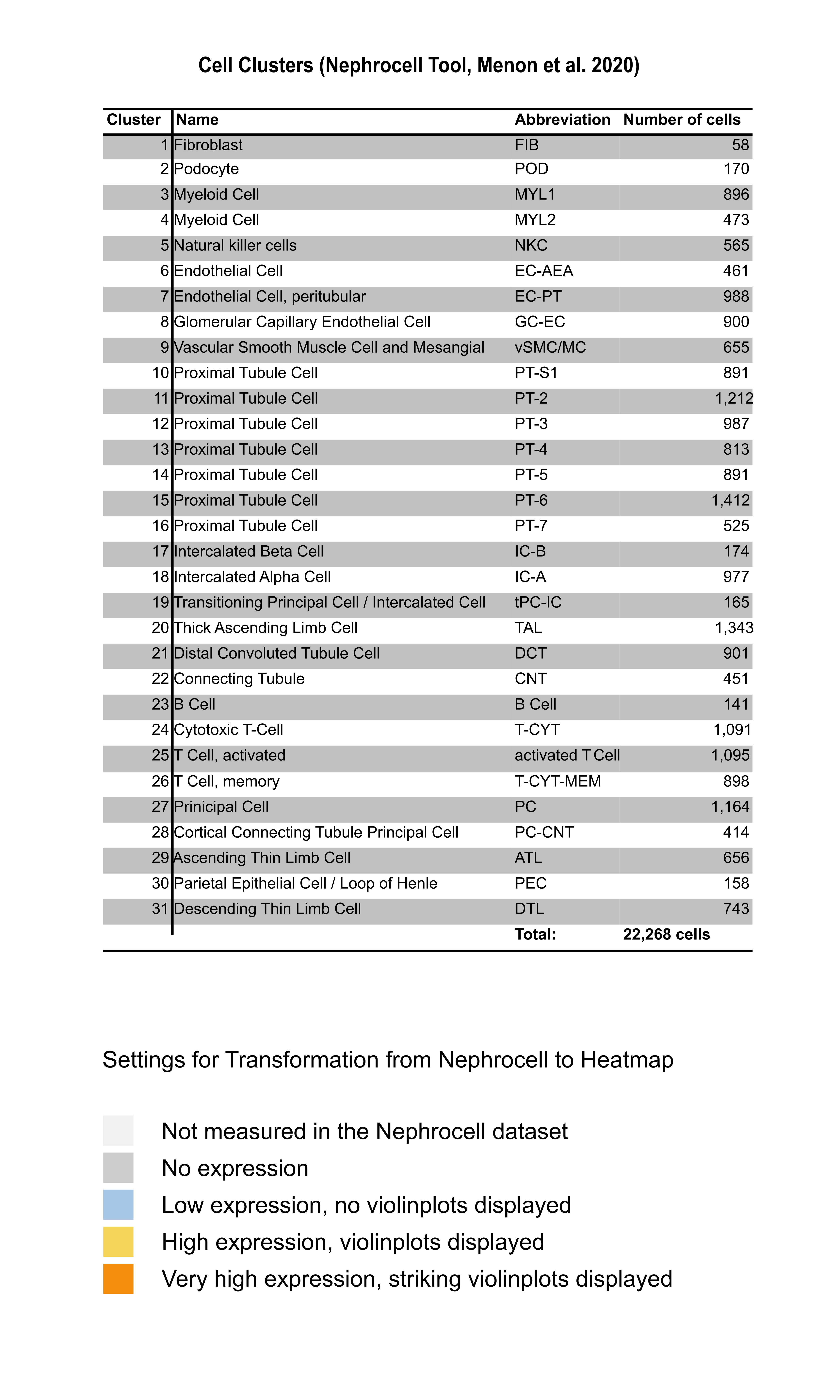
**

## **Figure S5:** Validation of CD_178_ genes in an independent *Pkd1^-/-^* mouse model.

**a)** For validation purposes, we performed RNA-seq on an independent Pkd1^-/-^ mouse model ^11^. Kidneys were obtained from 2 littermate control and 5 Pkd1^-/-^ mice (n = 7; Pkd1^tm2Ggg^/Pax8^rtTA^; induction from post-natal day 28 to 42; RNA isolation in week 12). RNA-seq data analysis in DESEQ2 yielded a total of 986 upregulated and 889 downregulated genes (FDR < 0.05). Due to fold changes differing from results in the cell culture model, no cut off for fold change was applied. **b)** Principal component analysis revealed principal component 1 (PC1) as discriminating factor between wild-type and Pkd1^-/-^ mice. As expected, this full-kidney-model showed a larger heterogeneity between replicates than our purified cell lines. **c)** Overlapping results from **a)** and **b)** yielded in a total of 282 genes, with more genes upregulated (n = 211) than downregulated (n = 71). These 282 genes were used as additional input for the cross-species meta-analysis (**Figure 6** and **Figure S6**).

**
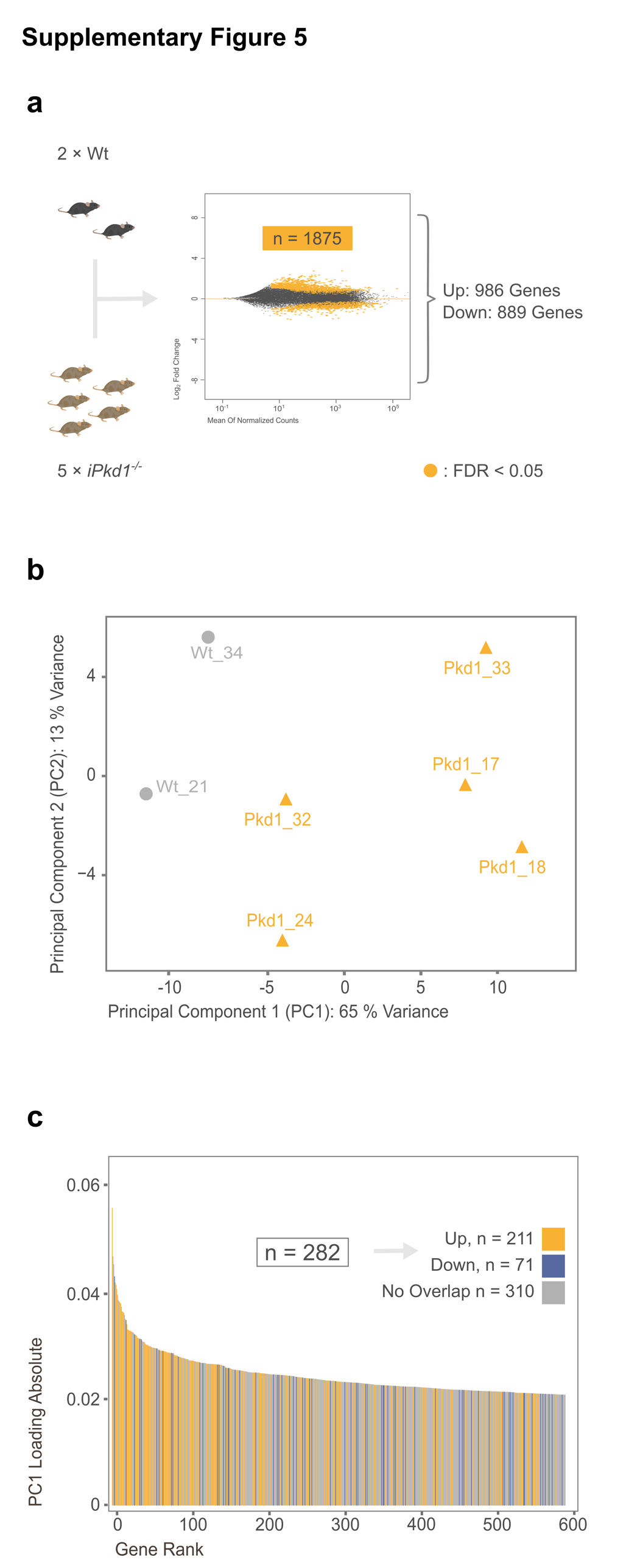
**

## **Figure S6:** CD_178_ genes were highly represented in other studies and elucidated novel functional cluster in ADPKD.

**a)** 9 independent PKD transcriptomes were analyzed for overlapping genes ^12-17^. 3 studies had been cross-matched before as Pkd1 Signature ^15^. Only genes represented in at least 2 of those 3 studies were used for our comparison. Thus, by selection, genes from ^13,15,16^ showed ≥ 2 overlaps. CD_178_ was the most consistent independent data set. CD_178_ replication across other studies was 64 %. Replication of other studies ranged from 20 % to 40 %. **b)** To integrate available data on PKD-responsive transcription in a meta-analysis, the minimum overlap between the CD_178_ and other studies was set to 3/8. 31 genes (18 %) met this criteria (Rep_31_). 61 genes were new to the core data set (New_61_). **c)** Up- and downregulation of Rep_31_ genes was model-dependent. 2 genes (APLN and PCSK9) were only up- and 4 genes (ALDH2, GAL3ST1, HS6ST2 and PANK1) only downregulated in all compared studies. 11 genes had only one study with a regulation opposing all other reported data. **d)** Functional clustering of CD_178_ genes identified three distinct clusters (**Figure 4c)**. Genes in Cluster A_10_ were highly represented in other studies, with 2 genes (CDH2 and TLR6) occurring in CD_178_ only and 31 total occurrences. Cluster B_8_ comprised 3 genes (CD34, DLX5 and HOXC9) uniquely identified in CD_178_ and 17 total occurrences. Cluster C_7_ had 3 genes (CSF2RA, PIK3R1 and PLCB1) occurring in CD_178_ only with a total of 12 occurrences.





# Supplementary Tables

## **Table S1:** Gene expression *Pkd1^-/-^* vs wildtype.

## **Table S2:** Gene expression *Pkd2^-/-^* vs wildtype.

## **Table S3:** DGE_254_

## **Table S4:** PCA_501_

## **Table S5:** CD_178_

## **Table S6:** Gene set enrichment analysis.

## **Table S7:** TF_7_

## **Table S8:** Published data sets for meta-analysis.

## **Table S9:** Y-chromosome

## **Table S10:** Gene set enrichment analysis – supplement.

# References

1. Hofherr A, Busch T, Huber N, et al. Efficient genome editing of differentiated renal epithelial cells. *Pflugers Arch.* 2017;469(2):303-311.

2. Afgan E, Baker D, Batut B, et al. The Galaxy platform for accessible, reproducible and collaborative biomedical analyses: 2018 update. *Nucleic Acids Res.* 2018;46(W1):W537-W544.

3. Radley AH, Schwab RM, Tan Y, Kim J, Lo EKW, Cahan P. Assessment of engineered cells using CellNet and RNA-seq. *Nat Protoc.* 2017;12(5):1089-1102.

4. Menon R, Otto EA, Hoover P, et al. Single cell transcriptomics identifies focal segmental glomerulosclerosis remission endothelial biomarker. *JCI Insight.* 2020;5(6).

5. Ransick A, Lindstrom NO, Liu J, et al. Single-Cell Profiling Reveals Sex, Lineage, and Regional Diversity in the Mouse Kidney. *Dev Cell.* 2019;51(3):399-413 e397.

6. Szklarczyk D, Gable AL, Lyon D, et al. STRING v11: protein-protein association networks with increased coverage, supporting functional discovery in genome-wide experimental datasets. *Nucleic Acids Res.* 2019;47(D1):D607-D613.

7. Schindelin J, Arganda-Carreras I, Frise E, et al. Fiji: an open-source platform for biological-image analysis. *Nat Methods.* 2012;9(7):676-682.

8. Love MI, Huber W, Anders S. Moderated estimation of fold change and dispersion for RNA-seq data with DESeq2. *Genome Biol.* 2014;15(12):550.

9. Karcher C, Fischer A, Schweickert A, et al. Lack of a laterality phenotype in Pkd1 knock-out embryos correlates with absence of polycystin-1 in nodal cilia. *Differentiation.* 2005;73(8):425-432.

10. Hateboer N, v Dijk MA, Bogdanova N, et al. Comparison of phenotypes of polycystic kidney disease types 1 and 2. European PKD1-PKD2 Study Group. *Lancet.* 1999;353(9147):103-107.

11. Viau A, Bienaime F, Lukas K, et al. Cilia-localized LKB1 regulates chemokine signaling, macrophage recruitment, and tissue homeostasis in the kidney. *EMBO J.* 2018;37(15).

12. Kunnen SJ, Malas TB, Formica C, Leonhard WN, t Hoen PAC, Peters DJM. Comparative transcriptomics of shear stress treated Pkd1(-/-) cells and pre-cystic kidneys reveals pathways involved in early polycystic kidney disease. *Biomed Pharmacother.* 2018;108:1123-1134.

13. Menezes LF, Zhou F, Patterson AD, et al. Network analysis of a Pkd1-mouse model of autosomal dominant polycystic kidney disease identifies HNF4alpha as a disease modifier. *PLoS Genet.* 2012;8(11):e1003053.

14. Chen WC, Tzeng YS, Li H. Gene expression in early and progression phases of autosomal dominant polycystic kidney disease. *BMC Res Notes.* 2008;1:131.

15. Malas TB, Formica C, Leonhard WN, et al. Meta-analysis of polycystic kidney disease expression profiles defines strong involvement of injury repair processes. *Am J Physiol Renal Physiol.* 2017;312(4):F806-F817.

16. Song X, Di Giovanni V, He N, et al. Systems biology of autosomal dominant polycystic kidney disease (ADPKD): computational identification of gene expression pathways and integrated regulatory networks. *Hum Mol Genet.* 2009;18(13):2328-2343.

17. Pandey P, Qin S, Ho J, Zhou J, Kreidberg JA. Systems biology approach to identify transcriptome reprogramming and candidate microRNA targets during the progression of polycystic kidney disease. *BMC Syst Biol.* 2011;5:56.
